# Patient priorities for fulfilling the principle of respect in research: findings from a modified Delphi study

**DOI:** 10.1186/s12910-023-00954-5

**Published:** 2023-09-21

**Authors:** Stephanie A. Kraft, Devan M. Duenas, Seema K. Shah

**Affiliations:** 1 Treuman Katz Center for Pediatric Bioethics and Palliative Care, Seattle Children’s Research Institute, 1900 Ninth Ave., M/S JMB-6, Seattle, WA 98101 USA; 2 Department of Pediatrics, University of Washington School of Medicine, Seattle, Washington USA; 3 Lurie Children’s Hospital, Chicago, IL USA; 4 Department of Pediatrics, Northwestern University Feinberg School of Medicine, Chicago, IL USA

**Keywords:** Research ethics, Respect for persons, Participant perspectives, Trustworthiness

## Abstract

**Background:**

Standard interpretations of the ethical principle of respect for persons have not incorporated the views and values of patients, especially patients from groups underrepresented in research. This limits the ability of research ethics scholarship, guidance, and oversight to support inclusive, patient-centered research. This study aimed to identify the practical approaches that patients in community-based settings value most for conveying respect in genomics research.

**Methods:**

We conducted a 3-round, web-based survey using the modified Delphi technique to identify areas of agreement among English-speaking patients at primary care clinics in Washington State and Idaho who had a personal or family history of cancer. In Round 1, respondents rated the importance of 17 items, identified in prior qualitative work, for feeling respected. In Round 2, respondents re-rated each item after reviewing overall group ratings. In Round 3, respondents ranked a subset of the 8 most highly rated items. We calculated each item’s mean and median rankings in Round 3 to identify which approaches were most important for feeling respected in research.

**Results:**

Forty-one patients consented to the survey, 21 (51%) completed Round 1, and 18 (86% of Round 1) completed each of Rounds 2 and 3. Two sets of rankings were excluded from analysis as speed of response suggested they had not completed the Round 3 ranking task. Respondents prioritized provision of study information to support decision-making (mean ranking 2.6 out of 8; median ranking 1.5) and interactions with research staff characterized by kindness, patience, and a lack of judgment (mean ranking 2.8; median ranking 2) as the most important approaches for conveying respect.

**Conclusions:**

Informed consent and interpersonal interactions are key ways that research participants experience respect. These can be supported by other approaches to respecting participants, especially when consent and/or direct interactions are infeasible. Future work should continue to engage with patients in community-based settings to identify best practices for research without consent and examine unique perspectives across clinical and demographic groups in different types of research.

**Supplementary Information:**

The online version contains supplementary material available at 10.1186/s12910-023-00954-5.

## Background

Genomics research has long failed to include participants representative of the full diversity of the global population, and marginalized and medically underserved groups remain underrepresented [1–3]. Diverse representation is ethically essential for ensuring that research is seen as inclusive and that research findings reflect patients’ lived experiences in the real-world settings where they receive care. The root causes of underrepresentation stem from and are upheld by longstanding, discriminatory attitudes, behaviors, and societal structures. Past research studies have actively and infamously harmed marginalized and minoritized individuals and communities, contributing to a legacy that researchers should be viewed with skepticism about whether they will fulfill their promises to minimize harms and maximize benefits [4–9]. Compounding this legacy of untrustworthy research, barriers such as time and transportation burdens of attending research visits, [10] reliance on preexisting clinical relationships for recruitment, [11] and limited outreach to patients in rural areas [12] systematically exclude groups of patients from participating.

Improving diversity in research therefore requires a multifaceted response that includes addressing access barriers and fostering collaborative partnerships through community engagement [13] and relationship building [14]. Part of this responsibility includes developing research approaches that respond to participants’ needs and values—including reevaluating how we define and operationalize the ethical principles that guide research. In particular, the principle of respect for persons is foundational to the ethical conduct of clinical research [15, 16] but is generally presumed to be fulfilled through regulatory measures to promote autonomous informed consent [17]. This narrow interpretation of respect for persons may not fully reflect the lived experiences and values of participants and potential participants, resulting in missed opportunities to fulfill the ethical obligation to respect each person as an individual and treat them how they wish to be treated. For example, one assessment of failed clinical trials identified elements that could make participants feel disrespected, such as long wait times for appointments or not being able to access study results (e.g., having to pay to access published articles) [18]. Broader approaches to examining researchers’ obligations to respect participants that examine the nuances of researcher-participant relationships may be better suited to incorporate these and other considerations by attending to participants’ lived realities and experiences of research [19].

To develop best practices for demonstrating respect in clinical research studies, especially as research initiatives increasingly strive to engage with underrepresented populations, it is essential to understand how individuals who have had little prior contact with research wish to be respected. Our prior work explored the perspectives of current genomics research participants on what activities contribute to their experiences of respect in research [20]. A critical next step is to examine patient views on respect among those who are not enrolled in genomics research and receive care in settings where they are unlikely to have had many opportunities to enroll, e.g., in community-based clinics. In this study, we built on our prior exploratory work and incorporated the views of patients in community-based settings about which activities should be prioritized in the development of interventions to improve potential genomics research participants’ experience of respect. We developed a web-based survey using a modified Delphi technique to identify areas of agreement among a panel of patient-respondents on activities that would be most important to demonstrate respect in research. We specifically sought to engage with individuals who would be potentially eligible to participate in genomics research but had not previously been invited to do so, to ensure approaches to respecting potential participants reflect the viewpoints of individuals who have been traditionally excluded from research.

## Methods

### Overview

We conducted a 3-stage modified Delphi survey in which patients rated and ranked approaches that would be most important to their experience of respect in genomics research [Supplemental Material]. The modified Delphi technique is a method used to develop consensus over time among an expert panel [21, 22]. For purposes of our research question, the relevant expert population is individuals who could be invited to participate in research but are not currently engaged in it—in this case, patients who would potentially be eligible to take part in a genomics research study due to personal or family history. While Delphi methods are often used to develop consensus among experts with professional expertise on the topic of interest, we sought to emphasize patients’ lived expertise in this study, and we used an asynchronous survey approach to maximize inclusion. Respondents were instructed that each round of the survey would build on the previous rounds and would incorporate their and others’ responses. This study was reviewed and approved by the Seattle Children’s Hospital Institutional Review Board.

### Recruitment

We recruited patients at three primary care clinics in western Washington, southwest Washington, and southern Idaho. Clinics were selected to include patients from rural and urban geographic areas and to reach people with diverse racial, ethnic, and socioeconomic backgrounds who were unlikely to have previously participated in research. Interested clinics were identified through the WWAMI region Practice and Research Network (WPRN), which is a primary care practice-based research network of clinics and clinical organizations in the 5-state WWAMI (Washington, Wyoming, Alaska, Montana, and Idaho) region [23]. Within the two states where we recruited, Washington and Idaho, in the 2020 US Census the median reported income was $82,4000 and $63,777, respectively, and the proportion of white-identifying individuals was 65% and 92%, respectively [24]. Patients were eligible to participate if they were age 18 + , could read and write English or Spanish, and reported any personal or family history of cancer. At one clinic, we identified potentially eligible patients with a personal or family history of cancer via an electronic health record query, mailed letters, and followed up by phone. At the other two clinics, we posted flyers, collected contact information from interested patients, and followed up by phone or email.

Because each round of the survey would build on previous rounds, including the possibility of new items based on the wording of respondents’ open-ended responses on the previous survey, we administered two series of surveys, one in English and one in Spanish. Each survey began with identical information, with the Spanish version translated by a certified translator and reviewed by a bilingual survey researcher with expertise in the Delphi method; subsequent survey rounds were modified based on intermediary responses, as described below. However, due to low recruitment and, consequently, inadequate sample size in the Spanish version, we only report here on the English version of the survey. The Spanish version of Round 1 is included in the Supplemental Material for reference.

**Fig. 1:**
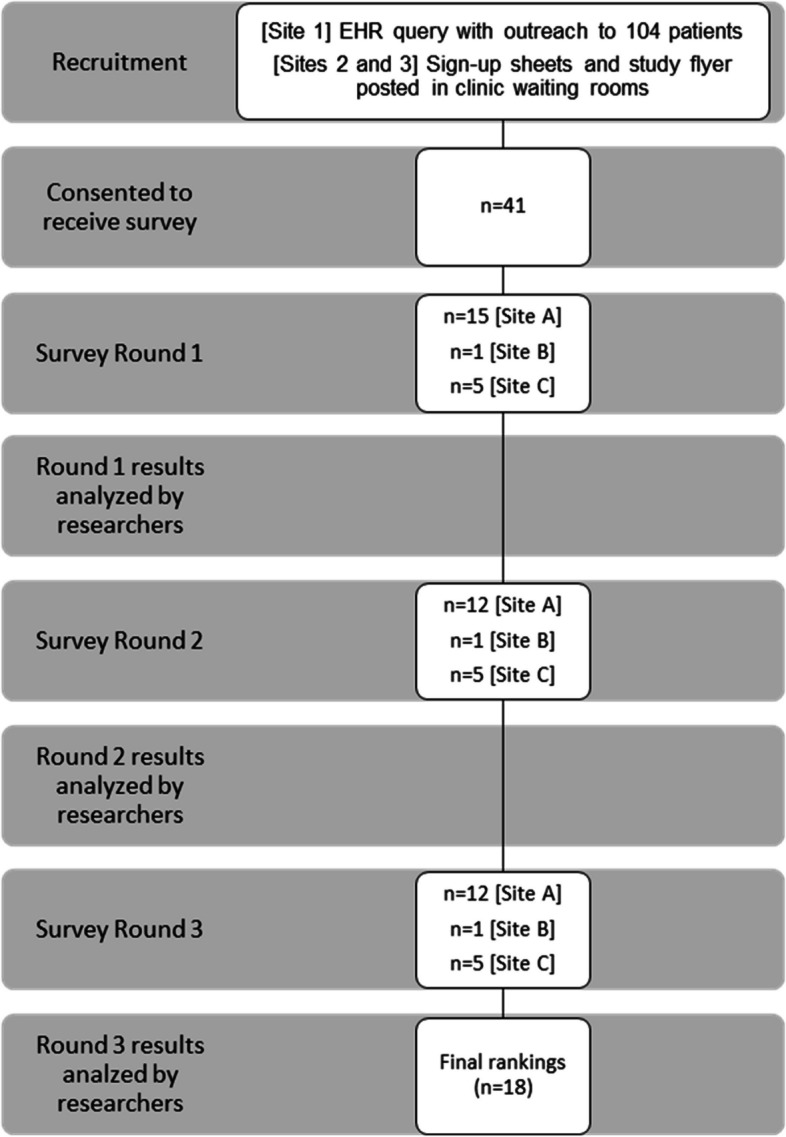
Recruitment flow chart

A total of 41 English-speaking individuals consented to participate and were sent an invitation to complete the first survey. Surveys were all completed via Qualtrics. Figure 1 shows recruitment and retention through the three survey rounds.

### Round 1

Round 1 of the survey asked respondents to rate a list of 17 items, which were developed based on our prior in-depth qualitative work that described genomics research participants’ perspectives on activities that convey respect in research [20]. Our prior work took place in the context of the implementation of a hereditary cancer screening program [25]. Because we anticipated that most respondents to this survey would not have prior research experience, we included a brief description of a hypothetical genomics research study involving screening and testing for hereditary cancer syndromes, similar to the study on which our prior work was based, to ground our respondents’ answers [Supplemental Material]. Following the research study description, respondents were asked to rate, on a Likert scale from 1 (not at all) to 5 (extremely) the importance of each of the 17 items for feeling respected in research. Respondents were given the opportunity to expand on their responses and to add any additional items of importance in open-ended responses. After rating the 17 items, respondents were asked additional questions about their experiences in clinical care and demographics. Round 1 was fielded from January to February 2021 and respondents received a $40 gift card.

### Round 2

Round 2 repeated the research study description from Round 1, followed by an opportunity to re-rate each of the 17 items from Round 1. For each item, respondents were shown a bar graph of all respondents’ responses to that item, reminded of how they had rated it, and asked to re-rate it. In addition, new items were added based on the open-ended responses from Round 1. These were identified through a two-coder process through which each coder assessed the clarity, scope, and uniqueness of each open-ended response. Those that met these criteria were reworded as needed and included as additional items after the first 17 items. Using this approach, we added 3 items. Round 2 was fielded in February 2021 and respondents received a $50 gift card.

### Round 3

We selected items for Round 3 using a categorical cut point based on responses to Round 2 such that the list of items in Round 3 would be feasible for respondents to complete the ranking task, which we evaluated through pilot testing. In determining our cut points, our goal was to identify approaches that were most important to the most people, which we determined post hoc to ensure we could meet this goal while maintaining a feasible list. We identified 8 items that 90% of respondents rated as “very” or “extremely” important on Round 2. In Round 3, respondents were again shown the research study description, then were asked to rank this subset of items from most to least important for feeling respected in research. At the end of the Round 3 survey, respondents were given the opportunity to respond to an open-ended question with any additional thoughts about their rankings or their experiences taking the surveys. Round 3 was fielded in March 2021 and respondents received a $60 gift card.

### Analysis

We calculated descriptive statistics for each item on each of the three surveys for each language group. We sorted the ranked items on Round 3 in order by mean ranking to produce our final list of respondent rankings. Two authors reviewed each response to the open-ended question on Round 3 to identify responses that expanded on the information provided in the closed-ended questions or added context to survey responses.

## Results

### Respondent characteristics

Of 41 respondents who consented to receive the survey, 21 (51%) completed Round 1. Of the 21 respondents to Round 1, 18 also responded to both Rounds 2 and 3 (86%). Most respondents (71% of Round 1, 67% of Rounds 2/3) were recruited from Site A. Additional respondent characteristics are shown in Table 1.

**Table 1:** Respondent characteristics

	**Round 1 (*n* = 21, 100%)**	**Rounds 2 and 3 (*n* = 18, 86% retention from Round 1)**
**Site**		
A	15 (71%)	12 (67%)
B	1 (5%)	1 (6%)
C	5 (24%)	5 (28%)
**Prior research experience**		
Yes	4 (19%)	2 (11%)
No	13 (62%)	12 (67%)
Not sure or don’t remember	4 (19%)	4 (22%)
**Age**		
18–24	2 (10%)	2 (11%)
25–34	3 (14%)	3 (17%)
35–44	7 (33%)	5 (28%)
45–54	2 (10%)	2 (11%)
55–64	3 (14%)	2 (11%)
65–74	3 (14%)	3 (17%)
75 or older	1 (5%)	1 (6%)
**Gender identity**		
Male	3 (14%)	2 (11%)
Female	16 (76%)	15 (83%)
Other: Trans man, Terran	2 (10%)	1 (6%)
**Race/ethnicity**		
Asian	2 (10%)	2 (11%)
Black or African American	4 (19%)	3 (17%)
White or European American	11 (52%)	11 (61%)
Hispanic/Latino(a)	1 (5%)	1 (6%)
Selected > 1 category	2 (10%)	1 (6%)
Unknown/none of these fully describe me	1 (5%)	0 (0%)
**Annual household income**		
Less than $20,000	6 (28%)	5 (28%)
$20,000 to $39,999	7 (33%)	5 (28%)
$40,000 to $59,999	2 (10%)	2 (11%)
$60,000 to $79,999	0 (0%)	0 (0%)
$80,000 to $99,999	3 (14%)	3 (17%)
$100,000 to $139,999	1 (5%)	1 (6%)
$140,000 or more	2 (10%)	2 (11%)
**Highest level of education**		
High school diploma or the equivalent such as GED	2 (10%)	2 (11%)
Trade or vocational school such as Beauty School or Electrical School	3 (14%)	2 (11%)
Some college	4 (19%)	3 (17%)
Associate’s degree or a two-year college degree	2 (10%)	2 (11%)
Bachelor’s degree or a four-year college degree	5 (24%)	4 (22%)
Master’s degree	3 (14%)	3 (17%)
Advanced degree such as a PhD, a Law degree, or a Medical degree	2 (10%)	2 (11%)
**Health insurance**		
Yes, public insurance, including Medicaid, Medicare, or other government-based plans	11 (52%)	9 (50%)
Yes, private insurance, including employer-based, direct-purchased, or TRICARE or other military insurance	8 (38%)	7 (39%)
Yes, other	1 (5%)	1 (6%)
Unknown	1 (5%)	1 (6%)
**Likelihood of joining a cancer research study**		
Not at all or a little likely	1 (5%)	1 (6%)
Somewhat likely	6 (29%)	6 (33%)
Very or extremely likely	14 (67%)	11 (61%)

### Rounds 1 and 2: Interim ratings

Initial item ratings in Round 1 and revised ratings in Round 2 are shown in Table 2.

**Table 2:** Full preliminary rankings from Rounds 1 and 2

	**Round 1 *n* = 21 (100%)**		**Round 2 *n* = 18 (87%)**	
	Mean (standard deviation)	Median (range; Q_1_, Q_3_)	Mean (standard deviation)	Median (range; Q_1_, Q_3_)
**To feel respected as a research participant, how important would it be to you that the research staff…**				
thoroughly describe the research study so you can decide whether to join?	4.6 (0.5)	5 (4–5; 4, 5)	4.7 (0.5)	5 (4–5 4.25, 5)
explain in a neutral way why you might or might not want to join the research study?^a^	4 (0.9)	4 (2–5; 3, 5)	4 (0.9)	4 (3–5; 3.25, 5)
give you plenty of time to make a decision about whether or not to join the research study?	3.5 (1.3)	4 (1–5; 3, 5)	3.3 (1.3)	3.5 (1–5; 2.25, 4)
give you options about which parts of the research study you want to be part of?	3.9 (0.9)	4 (2–5; 3, 5)	3.8 (0.8)	4 (3–5; 3, 4)
protect the privacy of your information?	4.6 (0.7)	5 (3–5; 4, 5)	4.6 (0.7)	5 (3–5; 4.25, 5)
show kindness, patience, non-judgment, and interest in you as a person?^a^	4.7 (0.5)	5 (4–5; 4, 5)	4.7 (0.6)	5 (4–5; 5, 5)
heck in with you to make sure you understand what you would be asked to do in the research study?^a^	4.4 (0.7)	5 (3–5; 4, 5)	4.6 (0.5)	5 (4–5; 4, 5)
show appreciation for your contributions to the research?	3.9 (1)	4 (2–5; 3, 5)	3.7 (0.8)	4 (3–5; 3, 4)
explain the benefits of the research study for you?	4.2 (1)	5 (2–5; 3, 5)	4.4 (0.8)	5 (3–5; 4, 5)
explain the benefits of the research study for society?	4.5 (0.7)	5 (3–5; 4, 5)	4.4 (0.8)	5 (3–5 4, 5)
have a specific person you can contact with questions?^a^	4.6 (0.5)	5 (4–5; 4, 5)	4.7 (0.5)	5 (4–5; 4, 5)
offer multiple ways of getting in touch with them, such as phone or email?	4.3 (0.7)	4 (3–5; 4, 5)	4.2 (0.6)	4 (3–5; 4, 5)
provide timely reminders and follow-ups?^a^	4.2 (0.7)	4 (3–5; 4, 5)	4.2 (0.4)	4 (4–5; 4, 4)
give you the results of your genetic testing?^a^	4.7 (0.6)	5 (3–5; 4, 5)	4.8 (0.4)	5 (4–5; 5, 5)
tell you about the overall research study findings?^a^	4.5 (0.6)	5 (3–5; 4, 5)	4.6 (0.6)	5 (4–5; 4, 5)
share the results of your genetic testing with your healthcare provider?	3.7 (1.3)	4 (1–5; 3, 5)	3.9 (0.9)	4 (2–5; 3.25, 4.75)
**To feel respected as a participant in the kind of cancer research described at the beginning of this survey, how important would it be to you that the study…**				
have research staff or interpreters who speak your language?^a^	4.4 (0.9)	5 (2–5; 4, 5)	4.6 (0.8)	5 (4–5; 4, 5)
write all research information in a way that is easy to read and understand?	4.5 (0.7)	5 (3–5; 4, 5)	4.5 (0.7)	5 (3–5; 4, 5)
allow you to take part in the research without needing to come into the clinic?	3.7 (1.1)	3 (1–5; 3, 5)	3.5 (0.9)	3 (2–5; 3, 4)
offer support and accommodations for people of all abilities?	4.5 (0.6)	5 (3–5; 4, 5)	4.4 (0.7)	4.5 (3–5; 4, 5)
**Survey 2 Exclusive Questions**				
To feel respected as a participant in the kind of cancer research described at the beginning of this survey, how important would it be to you that the study give you written materials on why the study is needed and important?	-	-	4.1 (0.7)	4 (3–5; 4, 5)
To feel respected as a participant in the kind of cancer research described at the beginning of this survey, how important would it be to you to have the option to speak with study staff in person?	-	-	3.7 (0.9)	4 (2–5 3, 4)
To feel respected as a participant in the kind of cancer research described at the beginning of this survey, how important would it be to you that you get to decide if your study test results are shared with your healthcare provider?	-	-	3.9 (1.1)	4 (2–5; 3, 5)

^a^Items that were included in Round 3 ranking task

### Round 3: Final rankings

In Round 3, respondents (*n* = 16, excluding 2 who did not spend sufficient time on the page to complete the ranking task) ranked “research staff thoroughly describe the research study so I can decide whether to join” (mean ranking 2.6 out of 8 (standard deviation 2.1); median ranking 1.5) and “research staff show kindness, patience, non-judgment, and interest in me as a person” (mean ranking 2.8 (standard deviation 1.8); median ranking 2) as the most important approaches for feeling respected, following by having a specific person to contact with questions (mean ranking 4.3 (standard deviation 1.8); median ranking 4), checking in to ensure understanding (mean ranking 4.3 (standard deviation 2.1); median ranking 4), and having research staff or interpreters who speak one’s language (mean ranking 4.7 (standard deviation 2.7); median ranking 4.5). Two items that were included in Round 3 but ranked relatively lower were “research staff give me the results of my genetic testing” (mean ranking 5.3 (standard deviation 1.6); median ranking 5.5) and “research staff tell me about the overall research study findings” (mean ranking 5.9 (standard deviation 1.7); median ranking: 5.5). Full rankings are shown in Table 3.

**Table 3:** Round 3 final rankings

Respondents (*n* = 18; rankings based on *n* = 16 who spent > 1 min on the ranking task) rated 8 items from 1 = most important to 8 = least important		
***To feel respected as a research participant, it would be important to me that the…***	**Mean ranking (standard deviation)**	**Median ranking (range; Q**_**1**_**, Q**_**3**_**)**
research staff thoroughly describe the research study so I can decide whether to join	2.6 (2.1)	1.5 (1–7; 1, 3.5)
research staff show kindness, patience, non-judgment, and interest in me as a person	2.8 (1.8)	2 (1–7; 1, 4)
research staff have a specific person I can contact with questions	4.3 (1.8)	4 (1–8; 3, 6)
research staff check in with me to make sure I understand what I would be asked to do in the research study	4.3 (2.1)	4 (2–8; 2.75, 5.25)
study have research staff or interpreters who speak my language	4.7 (2.7)	4.5 (1–8; 2, 8)
research staff give me the results of my genetic testing	5.3 (1.6)	5.5 (3–8; 4, 7)
research staff tell me about the overall research study findings	5.9 (1.7)	5.5 (2–8; 5, 7.25)
research staff provide timely reminders and follow-ups	6.1 (1.4)	6.5 (3–8; 5.75, 7)

Seven respondents provided additional detail in open-ended responses (excluding those who wrote “none,” “n/a,” or other similar responses). One respondent noted that they would rank some items equally. Two described items related to providing information or being kind as basic or “obvious” requirements, and one added that they would not participate in a study that did not fulfill these requirements. Others reiterated the importance of understanding study goals and how information will be used, expressed the importance of written information, and suggested edits to existing items (e.g., separating interest in a person from other aspects of personal study team interactions). One added that language was not a major concern for them given their perspective as an English speaker. Finally, one respondent described the importance of researchers being aware of their participants’ clinical context:

“Genetics, the experience of cancer in the family or oneself, and the risk of cancer are incredibly personal and emotional topics. After completing this sequence of surveys, I believe that holding space for the whole individual experience of disease and illness, as well the potential of our own illnesses (and impacts on our families), is the most important part of feeling respected in such a study.”

## Discussion

Our survey respondents collectively identified several activities that would be most important for their experience of respect in a hypothetical genomics study involving screening and testing for hereditary cancer syndromes. Items related to informed consent and study team interactions characterized by kindness, patience, and non-judgment were ranked as the most important respect-promoting activities. The high ranking of these items may reflect the fact that these interactions are often the most visible parts of a research study from a participant’s perspective, and thus a key locus for participants to experience respect, but it is also important for researchers to be aware how systems-focused approaches may shape these and other research experiences. Other highly ranked items on our survey may be particularly critical for showing respect in studies where consent and/or personal interactions are not feasible—for example, in some pragmatic clinical trials where varied considerations have been proposed that could, individually and/or collectively, contribute to respectful research [26]. Our findings also highlight a need for further work examining perceptions of respect among individuals who use languages other than English, as well as to support the development of respect-promoting interventions.

Our findings emphasize the importance of the informed consent process, as well as positive, informative, non-judgmental, and consistent interactions with research staff, for research participants to feel respected. These findings support the traditional understanding of consent as a central activity through which a research team can demonstrate respect for potential participants, highlighting the importance of both transparency about the study and control over one’s decision as key functions of a respectful consent process [27, 28]. They also highlight, as one respondent pointed out in an open-text response, that feeling respected in these ways may almost serve as a requirement for some people to participate in research and thus it is likely worth seeking ways to fulfill at least some functions of consent, such as providing transparency, even if full informed consent is impracticable. There also may be opportunities to further improve participants’ experiences of respect by ensuring the consent process meets individuals’ needs and supports their decision-making, for example through tailored or dynamic consent processes that are responsive to participant preferences and values [29, 30]. In doing so, researchers should not assume their responsibilities to respect participants end with promoting autonomy, but should incorporate attention to participants’ lived experiences and the full range of their needs and values.

Our findings also emphasize the importance of the research staff who are interacting with potential participants throughout the recruitment and consent processes. As one respondent wrote, “holding space for the whole individual experience of disease and illness” is critical for conveying respect, particularly in a setting where participants and their families may be experiencing serious illness. These findings build on our prior qualitative work in which participants identified personal interactions as a key way through which they perceived respect in research [20] and reflect the importance of the researcher’s contextual awareness and humility about participants’ lived experiences. Prior work has also identified positive and non-judgmental attitudes as facilitators for recruitment and retention, [31] illustrating how respectful interactions may be linked with enrollment decisions. Taken alongside the existing literature, our findings provide further evidence of the importance of training in and development of interpersonal skills, appropriate time and resources, and integration within the study to ensure research staff are well-positioned to engage with potential participants and address their questions and concerns [32–35].

Importantly, however, not all research studies involve direct personal interactions between potential participants and members of the research team. To ensure participants feel respected in such studies, researchers should consider how they might use remote research approaches or asynchronous interactions to complete the activities that were highly ranked by our respondents. For example, a web-based module can include a thorough explanation of a study, with additional detailed information available for those who are interested in learning more and checks for understanding throughout. Likewise, a specific contact person could be identified. Lower-ranked items from our final survey round may also reveal approaches to showing respect outside of the consent process or any interpersonal interactions, for example through providing high-quality information in the participant’s language, individual or overall study results, and/or other follow-ups. These may be particularly important to implement in the setting of pragmatic trials or other settings where consent is not feasible [26]. Given respondents’ comments that many of these approaches are seen as basic requirements, it may be important to strive to fulfill as many as possible within a study’s constraints. For example, while sharing genetic results and study findings have been discussed as approaches to respect participants [36, 37], the relatively lower rankings of these items suggest that, given the cost and complexity of sharing results, research teams could consider prioritizing investing in high-quality consent processes with easy-to-read written materials or hiring and training study coordinators who are skilled in engaging with participants respectfully. Future research could explore participants’ perspectives on how to navigate such trade-offs.

Further work is needed to identify priorities for demonstrating respect in different types of research with various populations. This study provided a high-level description of a hypothetical cancer genomics screening study, leaving the details open-ended, but participants’ perspectives may vary based on specific factors such as the clinical resources available to a participant to act on their results, whether and how screening is offered to family members, and how data are stored and shared. Other types of genomics and non-genomics studies may raise additional considerations that would shape participants’ priorities, as would studies focused on populations experiencing a particular health condition or lived experience. Future work should also develop and evaluate interventions to improve potential participants’ experiences of respect. Several interventions that our study points to as potential ways of improving the informed consent process have been implemented and evaluated to measure their impact on understanding, but not on the experience of respect. Our findings suggest that understanding and respect are interrelated and may need to be considered alongside one another. Interventions aiming to improve consent should consider a broader range of outcomes beyond understanding, recognizing that the consent process serves a range of functions, [27] including initiating and building a respectful research relationship.

## Limitations

Our study is limited by the relatively small number of our respondents and lack of substantial numbers of respondents who speak a language other than English. Despite our intentions to include Spanish-preferring respondents, we were unable to recruit a large enough sample to report, but we hypothesize that items such as the presence of interpreters or study staff who speak the participant’s language may be higher ranked among patients who prefer languages other than English. Still, the relatively high ranking of this item among our respondents suggests that patients see language accessibility as a key element of respectful research, even if they do not face language access barriers themselves. An additional limitation of this study is that our results may be biased due to the low response rate. While our respondents were diverse across several dimensions, they may be more supportive of research than others by virtue of having responded to this survey, and the most marginalized voices may not have come to the forefront. Future work should continue to examine whether individuals from different geographical regions, cultural backgrounds, and other socially identifiable groups prioritize different activities as conveying respect. For example, receiving research results may be particularly valuable for patients who lack meaningful clinical access to results, [11] and additional items related to proper use of personal pronouns may be important for gender-diverse individuals or others who regularly experience misgendering [38]. These and other experiences of individuals from marginalized social groups highlight the need to identify unique needs and values beyond those identified in our survey. Importantly, these and other items that were not ranked among the top respect-promoting activities may be critical for the experience of respect in certain settings or among certain groups, and even if not critical for respect, may have other ethical justifications. For example, sharing results with participants’ clinical providers may not be encompassed under the principle of respect, but may nevertheless be supported by the principle of beneficence. While our respondents prioritized autonomy-promoting approaches to respect based around interpersonal interactions, future work should examine more specifically how studies should enact other approaches to developing respectful procedures.

While the use of the modified Delphi technique allowed respondents to review each other’s answers and update their own responses over time, respondents may not have fully appreciated their role as part of a consensus panel, and the asynchronous design did not allow for interactive discussion that might have produced different results. We also note that the context in which this survey took place may have influenced our response rate as well as the responses of those who responded. Our recruitment and survey took place during a significant wave of the COVID-19 pandemic, which limited our recruitment in some clinics. This was also a time of intense public debate about equitable implementation of vaccinations that may have affected respondents’ views. While this context does not change the implications of our findings, it does highlight that participant preferences for how they are engaged in research may be context-dependent and that ongoing evaluation of the impact on participants is necessary.

## Conclusions

Participants in this study prioritized informed consent and interpersonal interactions as central to experiencing respect in research, alongside several other high priorities for a positive research experience. Future work should continue to explore participant perspectives on respect across different clinical populations, demographic groups, and research contexts, as well as to develop, implement, and evaluate tools and interventions to improve participants’ experiences of respect that build on these findings. Implementing such patient-centered approaches to respecting participants could enhance the ethics and inclusivity of research.

### Supplementary Information

**﻿Additional file 1:  Supplemental Material.** Survey instruments, Rounds 1-3 (English) and Round 1 (Spanish).

## Data Availability

The data generated and analyzed by this study are available from the corresponding author on reasonable request.

## Abbreviations

WPRN: WWAMI region Practice and Research Network
WWAMI: Washington, Wyoming, Alaska, Montana, and Idaho
