# Supplementary material for: Patient priorities for fulfilling the principle of respect in research: findings from a modified Delphi study
Source: BMC Med Ethics. 2023 Sep 21;24:73. doi: 10.1186/s12910-023-00954-5 (PMC10512546; doi:10.1186/s12910-023-00954-5)
Supplement: Supplementary file 1 — ﻿Additional file 1: Supplemental Material. Survey instruments, Rounds 1-3 (English) and Round 1 (Spanish). [file 12910_2023_954_MOESM1_ESM.pdf]

Supplemental Material. Survey instruments, Rounds 1-3 (English) and Round 1 (Spanish)

Note: Some survey instructions and page formatting instructions are omitted for publication. Images embedded in example study descriptions have been removed for publication.

Round 1, English

Welcome to the **Respect in Research** survey!

The **Respect in Research** survey is part of a research study. Our goal is to understand how people like to be treated in medical research.

This is the first of 3 surveys. In this survey, Survey 1, and the one to follow in a few weeks, Survey 2, we will ask you the same questions. In Survey 2, we will show you how you and everyone else taking part in the survey responded in Survey 1. You will be able to answer the same way you did in Survey 1 or change your answers. Finally, in Survey 3, we will show you the questions there was agreement on in Surveys 1 and 2 and ask you to rank them in order of how important they are to you.

Because each round of the survey builds on the round before it, no one can replace you in this study and we hope you will respond to every question in all 3 surveys.

Your answers to all rounds of the survey are confidential. No one participating in this study knows the names of the other participants, and this will be true during and after the study.

Survey 1 begins on the next page and will take about 30 minutes to complete. After you complete Survey 1, we will email you a $30 gift card as a thank you for your time.

This survey is about **how people like to be treated when they are asked to take part in a research study**. This page explains what might happen in a research study.

One type of research study involves patients who have had cancer or who have had cancer in their families. Some researchers study DNA, or genetics, because DNA can play a role in whether someone develops cancer. Genetic researchers study the role of DNA in cancer to improve how doctors take care of patients with a history of cancer in their families.

At the beginning of the study, researchers studying cancer in families would explain to patients what they would like to do in the research study. Then they would ask patients for permission to do it.

For example, researchers might ask patients to answer questions about their family history, give a saliva (spit) or blood sample for the researchers to use for genetic testing, and answer surveys or interviews. Researchers might ask to share patients' saliva or blood samples with other researchers. And researchers might ask to look at patients' medical records to learn about their overall health.

The researchers would also give patients contact information for their research staff so that patients could ask questions about the research study. Later, the researchers might give patients results from their genetic testing to tell them more about their health risks.

When answering the survey questions on the following pages, **please imagine you are being asked to join a research study like the one described above**.

The questions that follow are about what would be important to make you feel respected as a participant in the kind of cancer research study described on the previous page.

To feel respected as a research participant, how important would it be to you that the research staff ….

	Not at all important	A little important	Somewhat important	Very important	Extremely important
… thoroughly describe the research study so you can decide whether to join?					
… explain in a neutral way why you might or might not want to join the research study?					
… give you plenty of time to make a decision about whether or not to join the research study?					
… give you options about which parts of the research study you want to be part of?					
… protect the privacy of your information?					

Would you like to say more about your responses to any of the questions above? If yes, please tell us here:

To feel <u>respected</u> as a participant in the kind of cancer research described at the beginning of this survey, how <u>important</u> would it be to you that the research staff ….

	Not at all important	A little important	Somewhat important	Very important	Extremely important
… show kindness, patience, non-judgment, and interest in you as a person?					
… check in with you to make sure you understand what you would be asked to do in the research study?					
… show appreciation for your contributions to the research?					
… explain the benefits of the research study for you?					
… explain the benefits of the research study for society?					

Would you like to say more about your responses to any of the questions above? If yes, please tell us here:

To feel respected as a participant in the kind of cancer research described at the beginning of this survey, how important would it be to you that the research staff ….

	Not at all important	A little important	Somewhat important	Very important	Extremely important
… have a specific person you can contact with questions?					
… offer multiple ways of getting in touch with them, such as phone or email?					
… provide timely reminders and follow-ups?					
… give you the results of your genetic testing?					
… tell you about the overall research study findings?					
… share the results of your genetic testing with your healthcare provider?					

Would you like to say more about your responses to any of the questions above? If yes, please tell us here:

To feel respected as a participant in the kind of cancer research described at the beginning of this survey, how important would it be to you that the study ….

	Not at all important	A little important	Somewhat important	Very important	Extremely important
… have research staff or interpreters who speak your language?					
… write all research information in a way that is easy to read and understand?					
… allow you to take part in the research without needing to come into the clinic?					
… offer support and accommodations for people of all abilities?					

Would you like to say more about your responses to any of the questions above? If yes, please tell us here:

Is there anything else that would help you to feel respected as participant in the kind of cancer research described at the beginning of this survey that we did not ask about already? If so, please tell us below. Your answers to this question may be added to Survey 2 for all participants to consider.

Next, please tell us about your views on research and healthcare.

Thinking of the last interaction you had with a medical doctor or other healthcare professional, how respected did you feel in that interaction?

a. Not at all respected
b. A little respected
c. Somewhat respected
d. Very respected
e. Extremely respected

What made you feel [FILL: not at all\a little\somewhat\very\extremely] respected during the last interaction you had with a medical doctor or other healthcare professional? [open ended]

Thinking of how you are treated overall at the hospital or clinic you go to most often, how respected do you feel in those interactions?

a. Not at all respected
b. A little respected
c. Somewhat respected
d. Very respected
e. Extremely respected

What makes you feel [FILL: not at all\a little\somewhat\very\extremely] respected at the hospital or clinic you go to most often? [open ended]
How trustworthy would you say the doctor or healthcare professional you see most often is?

a. Not at all trustworthy
b. A little trustworthy
c. Somewhat trustworthy
d. Very trustworthy
e. Extremely trustworthy

What makes the doctor or healthcare professional you see most often [FILL: not at all\a little\somewhat\very\extremely] trustworthy? [open ended]

How trustworthy would you say the hospital or clinic you go to most often is?

a. Not at all trustworthy
b. A little trustworthy
c. Somewhat trustworthy
d. Very trustworthy
e. Extremely trustworthy

What makes the hospital or clinic you go to most often [FILL: not at all\a little\somewhat\very\extremely] trustworthy? [open ended]

Have you ever been in a medical research study before this survey?

a. Yes
b. No
c. Not sure or don’t remember

If you were invited to join a cancer research study like the one described at the beginning of this survey, how likely would you be to join it?

a. Not at all likely
b. A little likely
c. Somewhat likely
d. Very likely
e. Extremely likely

Finally, please answer the following optional, confidential questions about yourself.

How old are you?

a. 18-24
b. 25-34
c. 35-44
d. 45-54
e. 55-64
f. 65-74
g. 75 or older

How do you describe yourself?

a. Male
b. Female
c. Non-binary
d. Other __________

What category or categories best describe you? Check all that apply.

a. American Indian, Native American, or Alaska Native
b. Asian
c. Black or African American
d. Native Hawaiian/Pacific Islander
e. White or European American
f. Middle Eastern or North African/Mediterranean
g. Hispanic/Latino(a)
h. Unknown/none of these fully describe me

What was your household's total family income before taxes from all sources in 2020?

a. Less than $20,000
b. $20,000 to $39,999
c. $40,000 to $59,999
d. $60,000 to $79,999
e. $80,000 to $99,999
f. $100,000 to $139,999
g. $140,000 or more

What is the <u>highest</u> level of education you have <u>completed</u>?

a. 8th grade or less
b. Some high school
c. High school diploma or the equivalent such as GED
d. Trade or vocational school such as Beauty School or Electrical School
e. Some college
f. Associate's degree or a two-year college degree

g. Bachelor’s degree or a four-year college degree
h. Master’s degree
i. Advanced degree such as a PhD, a Law degree, or a Medical degree

Are you currently covered by any kind of health insurance?

a. No
b. Yes, public insurance, including Medicaid, Medicare, or other government-based plans
c. Yes, private insurance, including employer-based, direct-purchased, or TRICARE or other military insurance
d. Yes, other ________

Round 2, English

Welcome to Survey 2 of the **Respect in Research** study! This is the second of three surveys in this research study. Our goal is to understand how people like to be treated in medical research.

This survey has two sections. In the first section, we will ask the same questions we asked in Survey 1. We will show you how everyone, including you, answered each Survey 1 question. You are free to answer the same way you did in Survey 1, or you may change your answer after you see others' Survey 1 answers.

In section two, we will ask you questions that we created from answers to the following Survey 1 question: "Is there anything else that would help you to feel respected as a participant in the kind of cancer research described at the beginning of this survey that we did not ask about already?" The questions in section two will be new to you in this round.

Your responses are confidential. No one participating in this study knows the names of the other participants, and this will be true during and after the study.

In Survey 3, which we will send a few weeks after Survey 2 closes, we will show you what participants in Surveys 1 and 2 agreed is important to feel respected as a research participant, and we will ask you to rank your most important items.

Because each round of the survey builds on the round before it, no one can replace you in this study and we hope you will respond to every question in this and the next survey.

Survey 2 begins on the next page and will take about 30 minutes to complete. After you complete Survey 2, we will email you a $40 gift card as a thank you for your time.

[Note: Page repeating research description from Survey 1 omitted.]

Section 1.

The questions in this section are repeated from Survey 1.

[Note: The structure below is used for all Section 1 questions. The full list of Section 1 questions is below the structure.]

To feel respected as a participant in the kind of cancer research described at the beginning of this survey, **how important would it be to you that the … *[SEE FULL LIST OF SECTION 1 QUESTIONS BELOW]*?**

This is how you answered the question below in Survey 1:
[S1 RESPONSE]

The graph below shows how all participants, including you, answered the question above in Survey 1:

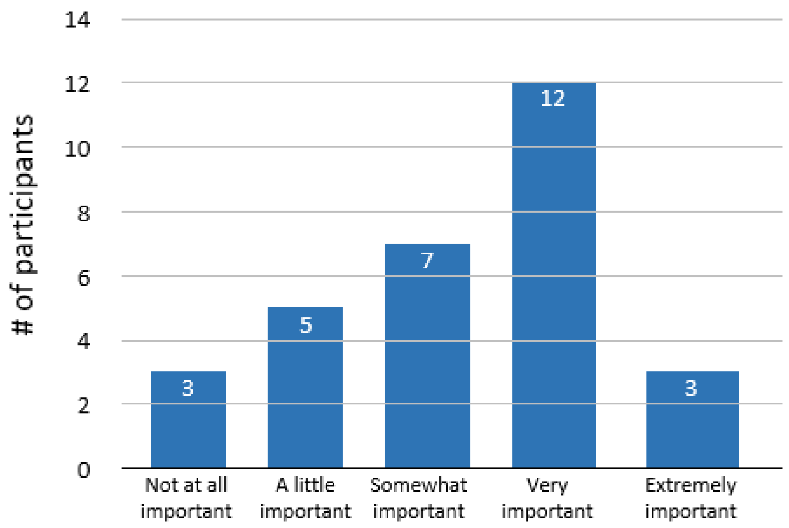

You are free to answer the question above the same way you did in Survey 1, or you may change your answer below based on other participants' Survey 1 answers.

- Not at all important
- A little important
- Somewhat important
- Very important
- Extremely important

FULL LIST OF SECTION 1 QUESTIONS

… research staff thoroughly describe the research study so you can decide whether to join?
… research staff explain in a neutral way why you might or might not want to join the research study?
… research staff give you plenty of time to make a decision about whether or not to join the research study?
… research staff give you options about which parts of the research study you want to be part of?
… research staff protect the privacy of your information?

… research staff show kindness, patience, non-judgment, and interest in you as a person?
… research staff check in with you to make sure you understand what you would be asked to do in the research study?
… research staff show appreciation for your contributions to the research?
… research staff explain the benefits of the research study for you?
… research staff explain the benefits of the research study for society?

… research staff have a specific person you can contact with questions?
… research staff offer multiple ways of getting in touch with them, such as phone or email?
… research staff provide timely reminders and follow-ups?
… research staff give you the results of your genetic testing?
… research staff tell you about the overall research study findings?
… research staff share the results of your genetic testing with your healthcare provider?

… study have research staff or interpreters who speak your language?
… study write all research information in a way that is easy to read and understand?
… study allow you to take part in the research without needing to come into the clinic?
… study offer support and accommodations for people of all abilities?

Section 2.

The questions below are new to Survey 2. They were not in Survey 1, so there are no Survey 1 answers to share.

To feel respected as a participant in the kind of cancer research study described at the beginning of this survey, **how important would it be to you that the study give you written materials on why the study is needed and important?**

- Not at all important
- A little important
- Somewhat important
- Very important
- Extremely important

To feel respected as a participant in the kind of cancer research study described at the beginning of this survey, **how important would it be to you to have the option to speak with study staff in person?**

- Not at all important
- A little important
- Somewhat important
- Very important
- Extremely important

To feel respected as a participant in the kind of cancer research study described at the beginning of this survey, **how important would it be to you that you get to decide if your study test results are shared with your healthcare provider?**

- Not at all important
- A little important
- Somewhat important
- Very important
- Extremely important

Would you like to say more about your responses to any of the questions above? If yes, please tell us here:

Round 3, English

Welcome to Survey 3 of the **Respect in Research** study! This is the third and final survey in this research study. Thank you for making it through to the last one!

In Surveys 1 and 2, you and the other participants told us what would be important to make you feel respected as a research participant. In this survey, we will ask you to rank the **top 8** things that would be **most important** to making you feel respected as a research participant.

Survey 3 begins on the next page and will take about 10 minutes to complete. After you complete Survey 3, we will email you a $50 gift card as a thank you for your time.

[Note: Page repeating research description from Survey 1 omitted.]

In Surveys 1 and 2, you and the other participants told us what would be important to make you feel respected as a research participant. We added up everyone's responses, and we show the top-8 most important statements below. The statements are in no particular order.

In this round, we would like you to rank the 8 statements below in order of how important they would be to making you feel respected as a participant in the kind of cancer research described on the previous page.

Please rank the statements below by "dragging" (clicking and moving) the most important statement to the top of the list. Then "drag" the next-most important statement to the second line in the list, and so on, so that all items are ranked from most important at the top to least important at the bottom.

If you are using a phone, it may be hard to see all 8 statements. Please be sure to scroll down so that you can consider and rank all 8.

All statements below begin here: **To feel respected as a research participant, it would be important to me that the...**

…research staff thoroughly describe the research study so I can decide whether to join
…research staff show kindness, patience, non-judgment, and interest in me as a person
…research staff check in with me to make sure I understand what I would be asked to do in the research study
…research staff have a specific person I can contact with questions
…research staff provide timely reminders and follow-ups
…research staff give me the results of my genetic testing
…research staff tell me about the overall research study findings
…study have research staff or interpreters who speak my language

[Note: Items will appear in random order using the Qualtrics response category randomizing function.]

Would you like to say anything about your rankings on the previous page or about any of your experiences over the 3 surveys for the Respect in Research study? If yes, please tell us here:

Round 1, Spanish

¡Bienvenidos a la encuesta **Respeto en la investigación**!

La encuesta **Respeto en la investigación** es parte de un estudio de investigación. Nuestro objetivo es comprender como las personas desean ser tratadas en la investigación médica.

Esta es la primera de las tres encuestas. Le haremos las mismas preguntas en esta encuesta, Encuesta 1, y en la que le seguirá en unas semanas, Encuesta 2. En la Encuesta 2, le mostraremos lo que usted y todas las personas que participan en la encuesta respondieron en la Encuesta 1. Podrá responder de la misma manera que lo hizo en la Encuesta 1 o cambiar sus respuestas. Finalmente, en la Encuesta 3, le mostraremos las preguntas sobre las que hubo acuerdo en las Encuestas 1 y 2 y le pediremos que las clasifique según el orden de importancia que tengan para usted.

Debido a que cada ronda de la encuesta se basa en la ronda anterior, nadie puede reemplazarle en este estudio y esperamos que responda todas las preguntas de las 3 encuestas.

Sus respuestas en todas las rondas de la encuesta son confidenciales. Nadie que participe en este estudio sabe los nombres de los demás participantes, y esto se mantendrá así durante y después del estudio.

La Encuesta 1 comienza en la página siguiente y le tomará unos 30 minutos completarla. Después de completar la Encuesta 1, le enviaremos por correo electrónico una tarjeta de regalo de $30 como agradecimiento por su tiempo.

Esta encuesta trata sobre **como las personas desean ser tratadas cuando se les pide que participen en un estudio de investigación.** Esta página explica lo que podría suceder en un estudio de investigación.

Un tipo de estudio de investigación involucra a pacientes que han tenido cáncer o que han tenido familiares con cáncer. Algunos investigadores estudian el ADN, o la genética, porque el ADN puede tener que ver con las posibilidades de desarrollar cáncer. Los investigadores genéticos estudian la participación del ADN en el cáncer para mejorar la atención de los médicos destinada a los pacientes con antecedentes de cáncer en sus familias.

Al comienzo del estudio, los investigadores que estudian el cáncer en familias les explicarían a los pacientes qué les gustaría hacer en el estudio de investigación. Luego les pedirían permiso a los pacientes para llevarlo a cabo.

Por ejemplo, los investigadores podrían pedirles a los pacientes que respondan preguntas sobre sus antecedentes familiares, que entreguen una muestra de saliva (escupa) o sangre para que los investigadores la utilicen en pruebas genéticas y que respondan a encuestas o entrevistas. Los investigadores podrían preguntar si pueden compartir con otros investigadores las muestras de saliva o de sangre de los pacientes. Y los investigadores podrían pedir ver el historial médico de los pacientes para conocer la salud de ellos en general.

Los investigadores también proporcionarían información de contacto de su personal de investigación a los pacientes para que los pacientes puedan hacer preguntas sobre el estudio de investigación. Luego, los investigadores podrían proporcionar a los pacientes los resultados de las pruebas genéticas para informarles más sobre los problemas de salud que tienen.

Cuando responda las preguntas de la encuesta en las páginas siguientes, **imagine que se le pide que participe en un estudio de investigación como el que se describe arriba.**

Las preguntas que están a continuación se refieren a lo que sería importante para que se sienta respetado como participante en el tipo de estudio de investigación del cáncer descrito en la página anterior.

Para sentirse respetado como participante en la investigación, ¿qué importancia tendría para usted que el personal de investigación…

	No importante en absoluto	Un poco importante	Algo importante	Muy importante	Extremadamente importante
... describa detalladamente el estudio de investigación para que usted pueda decidir si participa?					
... explique de una manera neutral el motivo por el qué usted podría o no participar en el estudio de investigación?					
... le dé tiempo suficiente para tomar una decisión sobre si participa o no en el estudio de investigación?					
... le brinde opciones sobre las partes del estudio de investigación en las que usted desea participar?					
... proteja la privacidad de su información?					

¿Le gustaría agregar otro comentario a sus respuestas a alguna de las preguntas anteriores? En caso afirmativo, díganos:

Para sentirse respetado como participante en el tipo de investigación del cáncer que se describe al principio de esta encuesta, ¿qué importancia tendría para usted que el personal de investigación…

	No importante en absoluto	Un poco importante	Algo importante	Muy importante	Extremadamente importante
... sea amable, paciente, no crítico y se interese por usted como persona?					
... le haga consultas para asegurarse de que usted comprende lo que se le pedirá que haga en el estudio de investigación?					
... le agradezca su contribución a la investigación?					
... le explique los beneficios del estudio de investigación para usted?					
... le explique los beneficios del estudio de investigación para la sociedad?					

¿Le gustaría agregar otro comentario a sus respuestas a alguna de las preguntas anteriores? En caso afirmativo, díganos:

Para sentirse respetado como participante en el tipo de investigación del cáncer que se describe al principio de esta encuesta, ¿qué importancia tendría para usted que el personal de investigación…

	No importante en absoluto	Un poco importante	Algo importante	Muy importante	Extremadamente importante
... tenga a una persona específica a la que usted pueda contactar si tiene preguntas?					
... ofrezca varias formas para ponerse en contacto con ellos, como por teléfono o correo electrónico?					
... proporcione recordatorios y seguimientos oportunos?					
... le entregue los resultados de su prueba genética?					
... le hable sobre los hallazgos generales del estudio de investigación?					
... comparta los resultados de su prueba genética con su proveedor de servicios médicos?					

¿Le gustaría agregar otro comentario a sus respuestas a alguna de las preguntas anteriores? En caso afirmativo, díganos:

Para sentirse respetado como participante en el tipo de investigación del cáncer que se describe al principio de esta encuesta, ¿qué importancia tendría para usted que en el estudio…

	No importante en absoluto	Un poco importante	Algo importante	Muy importante	Extremadamente importante
... haya personal de investigación o intérpretes que hablen su idioma?					
... se escriba toda la información de la investigación de una manera que sea fácil de leer y comprender?					
... se le permita participar en la investigación sin necesidad de acudir a la clínica?					
... se ofrezca apoyo y adaptaciones para personas de todas las capacidades?					

¿Le gustaría agregar otro comentario a sus respuestas a alguna de las preguntas anteriores? En caso afirmativo, díganos:

¿Hay algo más que le ayude a sentirse respetado como participante en el tipo de investigación del cáncer que se describe al principio de esta encuesta y que aún no le hemos preguntado? De ser así, díganos a continuación. Sus respuestas a esta pregunta pueden agregarse en la Encuesta 2 para que todos los participantes las consideren.

A continuación, cuéntenos lo que piensa sobre la investigación y la atención médica.

Si piensa en la última interacción que tuvo con un médico u otro profesional de la salud, ¿qué tan respetado se sintió en esa interacción?

a. No respetado en absoluto
b. Un poco respetado
c. Algo respetado
d. Muy respetado
e. Extremadamente respetado

¿Qué le hizo sentirse [FILL: no respetado en absoluto\un poco respetado\algo respetado\muy respetado\extremadamente respetado] durante la última interacción que tuvo con un médico u otro profesional de la salud? [preguntas abiertas]

Si piensa en cómo le tratan en general en el hospital o la clínica a la que acude con mayor frecuencia, ¿qué tan respetado se siente en esas interacciones?

a. No respetado en absoluto
b. Un poco respetado
c. Algo respetado
d. Muy respetado
e. Extremadamente respetado

¿Qué le hace sentirse [FILL: no respetado en absoluto\un poco respetado\algo respetado\muy respetado\extremadamente respetado] en el hospital o la clínica a la que acude con mayor frecuencia? [preguntas abiertas]

¿Qué tan confiable diría que es el médico o el profesional de la salud que visita con mayor frecuencia?

a. No confiable en absoluto
b. Un poco confiable
c. Algo confiable
d. Muy confiable
e. Extremadamente confiable

¿Qué hace que el médico o el profesional de la salud que visita con mayor frecuencia sea [COMPLETE: no confiable en absoluto\poco confiable\algo confiable\muy confiable\ extremadamente confiable? [preguntas abiertas]

¿Qué tan confiable diría que es el hospital o la clínica a la que acude con mayor frecuencia?

a. No confiable en absoluto
b. Un poco confiable
c. Algo confiable
d. Muy confiable
e. Extremadamente confiable

¿Qué hace que el hospital o la clínica a la que acude con mayor frecuencia sea [COMPLETE: no confiable en absoluto\poco confiable\algo confiable\muy confiable\extremadamente confiable? [preguntas abiertas]

¿Alguna vez participó en un estudio de investigación médica antes de esta encuesta?

a. Sí

b. No
c. No estoy seguro o no me acuerdo

Si se le invitara a participar en un estudio de investigación del cáncer como el que se describe al principio de esta encuesta, ¿qué tan probable es que participe?

a. No probable en absoluto
b. Un poco probable
c. Algo probable
d. Muy probable
e. Extremadamente probable

Por último, responda las siguientes preguntas opcionales y confidenciales sobre usted.

¿Qué edad tiene?

a. 18-24
b. 25-34
c. 35-44
d. 45-54
e. 55-64
f. 65-74
g. 75 o más

¿Cómo se describe a sí mismo?

a. Masculino
b. Femenino
c. No-binario
d. Otro __________

¿Qué categoría o categorías le describen mejor? Marque todas las que correspondan.

a. Indio estadounidense, nativo estadounidense o nativo de Alaska.
b. Asiático
c. Negro o afroamericano
d. Nativo de Hawái/islas del Pacífico
e. Blanco o europeo estadounidense
f. Del Medio Oriente o Norteafricano/mediterráneo
g. Hispano/Latino
h. Desconocido/ninguna de estas opciones me describen completamente

¿Cuál fue el ingreso total familiar en su hogar de todas las fuentes antes de los impuestos en 2020?

a. Menos de $20,000
b. De $20,000 a $39,999
c. De $40,000 a $59,999
d. De $60,000 a $79,999
e. De $80,000 a $99,999
f. De $100,000 a $139,999
g. $140,000 o más

¿Cuál es el nivel educativo <u>más alto</u> que ha <u>completado</u>?

a. 8.º grado o menor

b. Alguna escuela secundaria
c. Diploma de escuela secundaria o su equivalente como GED
d. Escuela comercial o vocacional como Escuela de Belleza o Técnico Electricista
e. Alguna universidad
f. Grado Técnico o título universitario de dos años
g. Licenciatura o título universitario de cuatro años
h. Maestría
i. Título superior, como un doctorado, un título en derecho o un título de médico

¿Está cubierto actualmente por algún tipo de seguro médico?

a. No
b. Sí, seguro público, incluidos Medicaid, Medicare u otros planes gubernamentales.
c. Sí, seguro privado, incluido el seguro del empleador, de compra directa o TRICARE u otro seguro militar
d. Sí, otro ________
